# High red blood cell distribution width is closely associated with in-stent restenosis in patients with unstable angina pectoris

**DOI:** 10.1186/s12872-019-1159-3

**Published:** 2019-07-24

**Authors:** Ning Geng, Guangsheng Su, Shaojun Wang, Deling Zou, Wenyue Pang, Yingxian Sun

**Affiliations:** 1 Department of Cardiology, Shengjing Hospital of China Medical University, No.36, Sanhao Street, Heping District, Shenyang City, Liaoning Province People’s Republic of China; 2 Department of Cardiology, The First Affiliated Hospital of China Medical University, Shenyang City, Liaoning Province China

**Keywords:** Unstable angina pectoris, in-stent restenosis, Percutaneous coronary intervention, Angiography, Red blood cell distribution width

## Abstract

**Background:**

In-stent restenosis remains an unresolved issue. Inflammation plays a pivotal role in the process of in**-**stent restenosis. Significant and positive associations were found between red blood cell distribution width (RDW) and inflammation. But whether there is a close relationship between higher RDW and in**-**stent restenosis is still not clarified.

**Methods:**

This retrospective observational study investigated 214 consecutive patients with unstable angina pectoris who underwent successful percutaneous coronary interventions with drug-eluting stents. Patients were divided into three groups according to baseline RDW before percutaneous coronary interventions (low RDW group:≤12.5%; intermediate RDW group:> 12.5% and ≤ 13.5%; high RDW group:> 13.5%). The follow-up angiographies were routinely performed 9–12 months after the initial percutaneous coronary interventions. The multivariate logistic regression analysis was employed to determine the independent predictors of in**-**stent restenosis.

**Results:**

The in**-**stent restenosis rate was significantly higher in group with higher baseline RDW value (12.3, 19.7, 47.7% in low, intermediate, and high RDW groups respectively, *P* < 0.001). The baseline RDWs were significantly higher in patients with in**-**stent restenosis compared with those in patients without in**-**stent restenosis (13.7 ± 0.8% vs. 13.0 ± 0.8%, *P* < 0.001). For prediction of in**-**stent restenosis, the ROC (receiver operating characteristic) curve analysis demonstrated the optimal RDW cutoff value was 13.37 (sensitivity: 65.5%, specificity: 73.6%); the diagnosis cutoff value was 13.89 (sensitivity: 40.0%, specificity: 91.8%); the screening cutoff value was 12.99 (sensitivity: 83.6%, specificity: 49.1%). By multivariate logistic analysis, higher baseline RDW (odds ratio [OR], 5.179; 95% confidence interval [CI], 2.568 to 10.446; *P****<***0.001) together with lower baseline indirect bilirubin (OR, 0.413; 95% CI, 0.305 to 0.559; *P****<***0.001) and diabetes (OR, 4.077; 95% CI, 1.654 to 10.054; *P* ***=*** 0.002) were closely associated with in**-**stent restenosis at followup (11.1 ± 5.8 months).

**Conclusions:**

The baseline RDW was closely associated with in**-**stent restenosis at follow-up. The patients with higher baseline RDW might have more chances to develop in**-**stent restenosis at followup.

## Background

Percutaneous coronary intervention (PCI) has become a promised therapy modality for coronary artery disease since 1979 [1], and drug-eluting stents (DES) have shown to significantly reduce the rate of in-stent restenosis (ISR) [2]. However, ISR has been encountered as a major limitation to the long-term efficacy of PCI even in the era of DES. In a meta-analysis of randomized controlled trials [3], the overall adjusted rate for angiographic restenosis was 10.5% in the DES group versus 31.7% in the control group (*P* < 0.001).

Although not completely elucidated, the inflammation is believed to play a critical role in the process of ISR [4]. The pathophysiology of ISR, which is characterized by neo-intimal hyperplasia, is an overreaction of the wound-healing response after the vascular injury caused by balloon dilation (barotrauma) and stent placement during PCI [5]. Vascular mechanical injury caused by PCI leads to substantial inflammation reaction which stimulates vascular smooth muscle cell proliferation and extracellular matrix deposition, resulting in neo-intimal thickening and restenosis [4]. Pre-interventional inflammatory status correlates with post-interventional adverse outcomes. Walter et al. found that higher tertiles of preprocedural C-type creative protein levels were independently associated with a higher risk of major adverse clinical events and angiographic restenoses after stenting [6].

The red blood cell distribution width (RDW) is a measurement of the size variation as well as an index of the heterogeneity of the erythrocytes. An increased RDW has been reported to be associated with increased inflammation status [7]. Higher RDW has been reported to be closely associated with adverse outcomes of cardiovascular disease such as heart failure [8] and prior myocardial infarction [9]. In this study, we explored whether the RDW before PCI was associated with ISR and could be used as an independent predictor of ISR after PCI in patients with unstable angina pectoris.

## Methods

### Patients and study design

This was a retrospective observational cohort study carried out at Shengjing hospital of China medical university. The consecutive unstable angina pectoris patients with coronary second generation, sirolimus eluting stent implantations and planned follow-up angiographies in our hospital from March 2015 to September 2015 were studied. All patient’s data were de-identified prior to data collection and the need for ethics approval was waived by our hospital. We excluded the patients with failed stenting; acute myocardial infarction; stable angina pectoris; major adverse cardiac events (death, myocardial infarction, stroke, coronary artery bypass graft, repeated PCI) after PCI; and patients with other conditions that can cause increased RDW (for example: anemia, heart failure, current acute infection, malignancy, chronic inflammatory disease and autoimmune disease) before the procedure and during the follow-up. The initial assessments of the patients included a medical history, physical examination, blood biochemistry tests for a complete blood cell count; total cholesterol, low-density lipoprotein cholesterol, high-density lipoprotein cholesterol and triglycerides; blood urea nitrogen; serum creatinine; nitrogen terminal-pro brain natriuretic peptide. The blood samples were drawn from participating patients after an overnight fast of more than 12 h in the morning of the initial PCI and were tested at the center for clinical chemistry of our hospital. The levels of RDW were measured with the use of the BECKMAN Ac. T 5 diff as a continuous variable. The normal range of RDW is 10.0–15.0 in our hospital.

### PCI procedures and follow-up angiography evaluations

Drug eluting stents were implanted in all patients according to the current guidelines. All patients received antiplatelet therapy before and after the procedures: 100 mg aspirin was continued indefinitely and 75 mg clopidogrel was continued for at least 1 year after stent implantation. The follow-up angiographies were routinely performed 9–12 months after the initial PCIs. The evaluations of restenosis were carried out using the conventional QCA technique. ISR at follow-up was defined as luminal narrowing of more than 50% occurring in the segment within the stent or within a 5 mm segment proximal or distal to the stent.

### Statistical analysis

IBM SPSS Statistics 23.0 was used to analyze the data. Categorical variables were reported as frequencies (percentage) and were compared using chi-square analysis. Continuous variables were reported as means±standard deviations and were compared by Student’s *t* test. A one-way analysis of variance was used to determine differences among different categories of RDW. Differences at a two tailed *P* value less than 0.05 were considered significant. Binary logistic regression analysis was carried out to assess the risk factors of ISR. Odds ratio (OR) and 95% confidence interval (CI) were calculated. Among the variables tested, only those variables with statistical significance set at *P* less than 0.05 at univariate analysis together with age and gender were included in a multivariate logistic regression model to determine the independent predictors of ISR. We also performed a receiver operating characteristics (ROC) curve analysis to determine the optimal cutoff, diagnostic cutoff, screening cutoff value of RDW for prediction of in-stent restenosis. The optimal cut-off value is the point on the curve that is closest to the top of the left hand *y*-axis. This is the point at which the true positive rate is optimized and the false positive rate is minimized. The screening cut-off value is chosen to maximize the sensitivity of the test while maintaining an appropriate speciality. The diagnostic cut-off value is chosen to maximize the specificity of the test while maintaining an appropriate sensitivity. The determinations of diagnostic cutoff and screening cutoff value are often an expert decision and subjective to some degree.

## Results

### General characteristics of the patients with in-stent restenosis and without in-stent restenosis

A total of 233 patients with unstable angina pectoris and drug eluting stent implantations were screened. Eleven patients lost follow-up; 2 patients died; 6 patients suffered heart failure during follow-up. So 214 patients were eligible for this study. After an average follow-up of 11.1 ± 5.8 months, 55 patients suffered in-stent restenosis. The baseline characteristics of these patients were summarized in Table 1. There were no differences in the follow-up period between the ISR group and non-ISR group. Diabetes were more common in patients with ISR. Patients with ISR were more likely to be older; had a higher baseline and followup RDW; and lower total bilirubin, direct and indirect bilirubin; had longer stents of smaller diameters implanted.

**Table 1:** Baseline characteristics of the patients included

Variable	Overall (*n* = 214)	Non-ISR (*n* = 159)	ISR (*n* = 55)	*P* value
Age (years)	63.3 ± 9.9	62.9 ± 9.7	64.4 ± 10.2	0.040
Male gender (%)	151 (70.1)	112 (70.4)	39 (70.9)	0.948
Follow-up period (months)	11.1 ± 5.8	10.8 ± 5.4	12.1 ± 7.1	0.164
Length of stents (mm)	26.8 ± 5.6	26.4 ± 6.0	28.0 ± 4.3	0.034
Diameter of stents (mm)	3.1 ± 0.4	3.1 ± 0.4	2.9 ± 0.3	0.014
Complex lesion^a^ (%)	91 (42.5)	65 (40.9)	26 (47.2)	0.408
Clinical variables				
Hypertension (%)	134 (62.6)	98 (61.6)	36 (65.5)	0.614
Diabetes mellitus (%)	68 (31.8)	37 (23.3)	31 (56.4)	<0.001
Laboratory results				
Total bilirubin (μmol/L)	12.4 ± 4.5	13.6 ± 4.4	8.9 ± 2.8	<0.001
Direct bilirubin (μmol/L)	4.5 ± 1.8	4.9 ± 1.8	3.4 ± 1.3	<0.001
Indirect bilirubin (μmol/L)	7.9 ± 2.9	8.7 ± 2.8	5.5 ± 1.7	<0.001
Baseline RDW (%)	13.1 ± 0.8	13.0 ± 0.8	13.7 ± 0.8	<0.001
RDW (%) at followup	13.4 ± 1.0	13.2 ± 0.8	14.0 ± 1.4	<0.001
Hb (g/L)	137.3 ± 15.0	138.0 ± 14.6	135.4 ± 16.3	0.270
Baseline WBC (10^9^/L)	7.7 ± 2.7	7.9 ± 2.8	7.3 ± 2.5	0.553
WBC at followup (10^9^/L)	6.8 ± 1.8	6.7 ± 1.7	7.0 ± 2.0	0.267
BUN (mmol/L)	6.0 ± 2.0	5.9 ± 1.9	6.3 ± 2.2	0.322
eGFR (ml/min)	84.6 ± 23.6	84.5 ± 22.8	85.2 ± 26.1	0.849
TG (mmol/L)	1.5 (1.2)*	1.6 (1.2)*	1.4 (1.3)*	0.476
HDL-C (mmol/L)	1.0 ± 0.3	1.0 ± 0.3	1.0 ± 0.3	0.553
LDL-C (mmol/L)	2.7 ± 1.0	2.7 ± 0.9	2.7 ± 1.2	0.867
Pharmacotherapy				
ACEI or ARB	142 (66.4)	108 (67.8)	34 (62.1)	0.570
ß-Blocker	70 (32.6)	51 (32.2)	19 (34.5)	0.819
Statin	186 (87.1)	139 (87.4)	47 (86.2)	0.873

Data are represented as number (%) or mean ± standard deviation

^a^Complex lesion refers to type B2 and C lesion

*: median (inter-quartile range)

*ACEI* Angiotensin-converting enzyme inhibitor, *ARB* Angiotensin receptor blocker, *BUN* Blood urea nitrogen, *eGFR* estimated glomerular filtration rate, *Hb* Hemoglobin, *HDL-C* High-density lipoprotein cholesterol, *ISR* In-stent restenosis, *LDL-C* Low-density lipoprotein cholesterol, *RDW* Red blood cell distribution width, *TG* Triglyceride, *WBC* White blood cell

### Comparison between the baseline RDWs and the follow-up RDWs

We compared the RDWs prior to the PCI (RDW_baseline_) with the RDWs at follow-up (RDW_follow-up_) and interestingly we found that the RDWs increased significantly during follow-up in both the non-ISR group (13.0 ± 0.8 vs. 13.2 ± 0.8; *P* = 0.003) and the ISR group (13.7 ± 0.8 vs. 14.0 ± 1.4 *p* = 0.022). The increases of RDW during follow-up were not significantly different between in non-ISR and ISR groups (*P* = 0.472). (Table 2).

**Table 2:** Comparison between the baseline RDW and the follow-up RDW

	RDW_baseline_ (Mean ± SD)	RDW_followup_ (Mean ± SD)	RDW change (Mean ± SD)	*P* value
Overall (%)	13.1 ± 0.8	13.4 ± 1.0	0.2 ± 0.8	<0.001
Non-ISR (%)	13.0 ± 0.8	13.2 ± 0.8	0.2 ± 0.8^a^	0.003
ISR (%)	13.7 ± 0.8	14.0 ± 1.4	0.3 ± 0.9^a^	0.022

^a^The difference of RDW changes between in Non-ISR and ISR groups is not statistically significant (*P* = 0.472)

*ISR* In-stent restenosis, *RDW* Red blood cell distribution width, *SD* Standard deviation

### Relationship between baseline RDW and the occurrence of ISR at follow-up

We divided the participants into three groups according to the tertile of RDW values before the initial PCI (low RDW group: ≤ 12.5%; intermediate RDW group: > 12.5% and ≤ 13.5%; high RDW group: > 13.5%) to explore the relationship between the baseline RDWs and the ISR at follow-up. The clinical and angiographic characteristics were compared among different RDW groups (Table 3). We found that the patients with higher baseline RDWs were more likely to develop ISR at follow-up (low RDW:12.3%; intermediate RDW:19.7%; high RDW:47.7%; *P* < 0.001). There were no statistically significant differences in clinical and laboratory characteristics among different RDW categories except age, stent diameter and hemoglobin.

**Table 3:** Clinical characteristics of different red blood cell distribution width categories

	Low RDW (*n* = 73)	Intermediate RDW (*n* = 76)	High RDW (*n* = 65)	*P* value
RDW (%)	12.3 ± 0.6	13.2 ± 0.2	14.1 ± 0.5	<0.001
Age (years)	64.2 ± 8.7	61.0 ± 10.7	64.9 ± 9.8	0.038
Fellow-up (month)	11.0 ± 7.0	11.1 ± 4.1	11.4 ± 6.2	0.931
Male gender	55 (75.3)	53 (69.7)	43 (66.2)	0.488
ISR (%)	9 (12.3)	15 (19.7)	31 (47.7)	<0.001
Stent diameter (mm)	3.1 ± 0.2	3.1 ± 0.4	2.9 ± 0,3	0.028
Stent length (mm)	26.2 ± 6.0	27.3 ± 5.3	27.0 ± 5.6	0.510
Complex lesion^a^ (%)	31 (42.5)	29 (38.2)	31 (47.7)	0.521
Clinical variables				
Hypertension	46 (63.0)	58 (76.3)	38 (58.5)	0.132
Diabetes mellitus	17 (25.0)	27 (39.7)	24 (35.3)	0.156
Laboratory results				
Total bilirubin (μmol/L)	12.4 ± 4.4	12.8 ± 4.7	12.0 ± 4.6	0.584
Direct bilirubin (μmol/L)	4.4 ± 1.7	4.6 ± 2.0	4.6 ± 1.9	0.586
Indirect bilirubin (μmol/L)	7.9 ± 2.9	8.2 ± 2.9	7.3 ± 3.0	0.200
Hb (g/L)	138.3 ± 14.1	140.9 ± 14.3	132.0 ± 15.6	0.002
WBC (10^9^/L)	7.7 ± 2.5	7.9 ± 3.1	7.6 ± 2.5	0.751
BUN (mmol/L)	5.9 ± 1.8	5.8 ± 1.8	6.3 ± 2.5	0.350
eGFR (ml/min)	85.6 ± 23.0	86.5 ± 23.6	81.4 ± 24.2	0.397
TG (mmol/L)	2.0 ± 1.3	1.9 ± 1.1	2.0 ± 2.1	0.963
HDL-C (mmol/L)	1.0 ± 0.2	1.0 ± 0.3	1.1 ± 0.3	0.257
LDL-C (mmol/L)	2.6 ± 0.8	2.8 ± 0.8	2.7 ± 1.2	0.524
Pharmacotherapy				
ACEI or ARB	50 (68.5)	52 (68.4)	40 (61.5)	0.616
β-Blocker	23 (31.5)	25 (32.9)	21 (32.3)	0.984
Statin	63 (86.3)	68 (89.5)	54 (83.1)	0.542

Patients were divided into three groups according to baseline RDW (low:≤12.5%; intermediate:> 12.5% and ≤ 13.5%; high:> 13.5%)

Data are represented as number (%) or mean ± SD

^a^: refers to type B_2_ and C lesions

*ACEI* Angiotensin-converting enzyme inhibitor, *ARB* Angiotensin receptor blocker, *BUN* Blood urea nitrogen, *eGFR* estimated glomerular filtration rate, *Hb* Hemoglobin, *HDL-C* High-density lipoprotein cholesterol, *ISR* In-stent restenosis, *LDL-C* Low-density lipoprotein cholesterol, *RDW* Red blood cell distribution width, *TG* Triglyceride, *WBC* White blood cell

### Predictors of ISR at follow-up

Binary logistic regression analysis was used to assess independent predictors of ISR at follow-up. All variables that were clinically important and potentially correlated with ISR were included in the univariate logistic model (Table 4). We found that diabetes, indirect bilirubin, baseline RDW, hemoglobin were the significant univariable predictors of the ISR. Then we applied multivariate logistic regression analysis that included all the significant univariable predictors found in Table 4 to determine the independent predictors of ISR. The odds ratio and *P* value were adjusted for age and gender (Table 5). In the multivariate analysis model, only the baseline RDW (OR, 5.179; 95% CI, 2.568 to 10.446, *P****<***0.001); indirect bilirubin (OR, 0.413; 95% CI, 0.305 to 0.559, *P****<***0.001) and diabetes (OR, 4.077; 95% CI, 1.654 to 10.054, *P* ***=*** 0.002) were significant independent predictors of ISR at follow-up.

**Table 4:** Univariable logistic regression analysis for the prediction of ISR at followup

Variables	OR	95% CI	*P* value
Age	1.008	0.941–1.079	0.817
Gender	0.620	0.163–2.356	0.483
Hypertension	1.781	0.542–5.853	0.342
Diabetes mellitus	3.583	1.122–11.441	0.031
LDL-C	0.738	0.384–1.419	0.363
HDL-C	0.879	0.090–8.625	0.912
TG	0.873	0.554–1.376	0.558
BUN	1.071	0.802–1.432	0.641
eGFR	1.006	0.975–1.037	0.724
Indirect bilirubin	0.363	0.220–0.599	<0.001
Direct bilirubin	1.004	0.549–1.836	0.988
Baseline RDW	3.903	1.375–11.075	0.011
Follow-up RDW	1.727	0.745–4.007	0.203
Hb	1.041	1.000–1.083	0.048
Baseline WBC	0.764	0.574–1.018	0.066
Followup WBC	1.322	0.920–1.901	0.131
Complex lesion^a^	1.142	0.346–3.764	0.828
Stent diameter (mm)	0.427	0.089–2.034	0.285
Stent length (mm)	1.075	0.965–1.197	0.191
ACEI or ARB	0.425	0.126–1.432	0.167
β-blocker	2.525	0.731–8.716	0.143
Statin	0.818	0.191–3.492	0.786

^a^Complex lesion refers to type B2 and C lesion

*ACEI* Angiotensin-converting enzyme inhibitor, *ARB* Angiotensin receptor blocker, *BUN* Blood urea nitrogen, *eGFR* estimated glomerular filtration rate, *Hb* Hemoglobin, *HDL-C* High-density lipoprotein cholesterol, *ISR* In-stent restenosis, *LDL-C* Low-density lipoprotein cholesterol, *RDW* Red blood cell distribution width, *TG* Triglycerides, *WBC* White blood cell

**Table 5:** Independent predictors of ISR at followup by multivariable logistic regression analysis

Variables	OR	95% CI	*P* value
Age	1.035	0.987–1.085	0.151
Gender	0.615	0.210–1.802	0.376
Baseline RDW	5.179	2.568–10.446	<0.001
Hb	1.029	0.995–1.064	0.093
Indirect bilirubin	0.413	0.305–0.559	<0.001
DM	4.077	1.654–10.054	0.002

*CI* Confidence interval, *Hb* Hemoglobin, *ISR* In-stent restenosis, *OR* Odds ratio, *RDW* Red blood cell distribution width

### ROC curve analysis

**Fig. 1:**
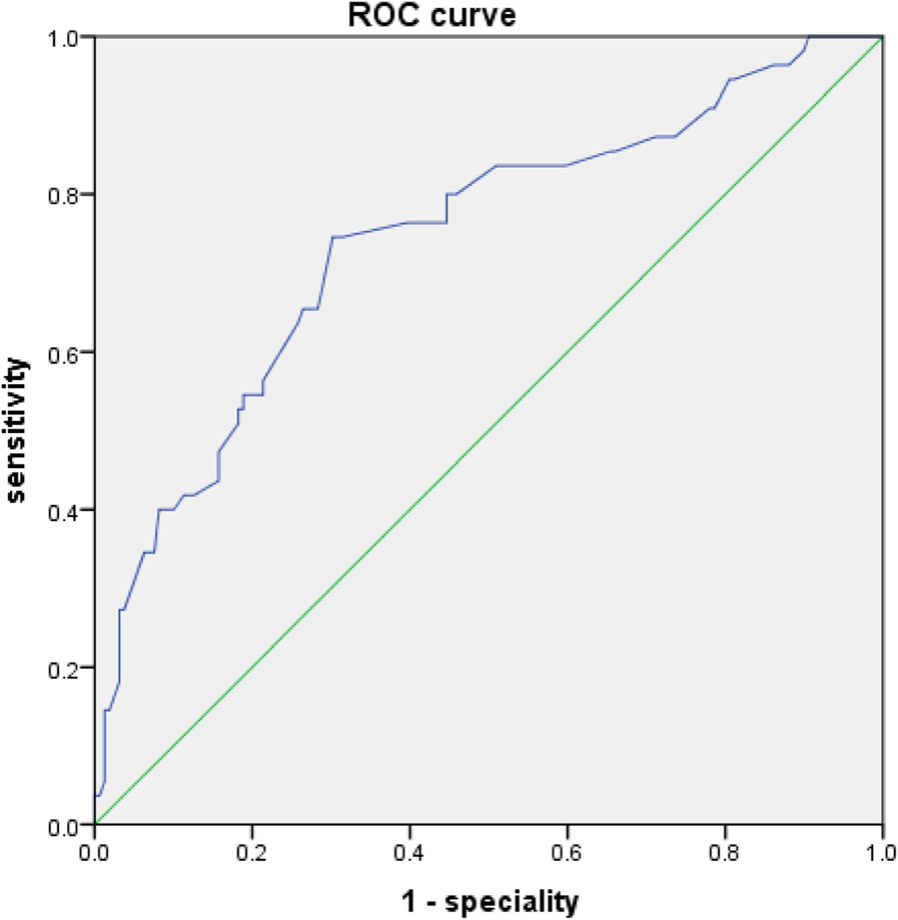
The ROC curve analysis of RDW for predicting ISR. ISR, in-stent restenosis; RDW, red blood cell distribution width; ROC, receiver operating characteristic

ROC curve analysis of the diagnostic accuracy of the RDW for in-stent restenosis showed an area under the curve (AUC) of 0.743 (95% CI, 0.664 to 0.822; *P****<***0.001) (Fig. 1), and the optimal cutoff value of RDW for discrimination of in-stent restenosis was 13.37 (sensitivity:65.5%, specificity:73.6%). The diagnostic cutoff value of RDW was 13.89 with a specificity 91.8% and sensitivity 40%. A RDW 12.99 was used for the screening cutoff value with a sensitivity 83.6% and specificity 49.1%.

## Discussion

In the present study, we found that the baseline RDW, indirect bilirubin and diabetes before PCI were strongly associated with the occurrence of ISR and might be used as independent predictors of ISR in patients with unstable angina pectoris at followup. The RDW is a measurement of the size variation as well as an index of the heterogeneity of the erythrocytes. Both the red blood cell destruction and decreased production can increase the value of RDW. So the RDW can be typically used in the differential diagnosis of underlying anemias [10]. However, several lines of evidences have recently attested the pathogenetic link between RDW and a lot of cardiovascular disorders. The increased RDW can serve as an independent predictor of mortality in patients with heart failure [8], coronary heart disease [9], and even in unselected general middle-aged and older adults [11].

In our study, we tried to evaluate the relationship between the higher RDW before PCI and the risk of ISR at follow-up. Although the exact pathophysiological mechanisms underlying the association between increased RDW and ISR are not fully understood, we believe that the inflammatory status, which can both alter erythrocyte homeostasis and affect the process of ISR, are likely to play an important role in the association between the baseline RDW and ISR at follow-up. The RDW can act as a marker of the inflammatory state in the body. In a series of clinical investigations, significant and positive associations were found between RDW and a variety of inflammatory markers, such as high sensitive C-creative protein and erythrocyte sedimentation rate [7], interleukin (IL)-6 [12], soluble transferrin receptor, soluble tumor necrosis factor (TNF) receptor I, soluble TNF receptor II [13]. Inflammation can contribute to an increased RDW by impairing iron metabolism, inhibiting the production of or response to erythropoietin, or by shortening red blood cell survival [14] and it has been demonstrated that inflammatory cytokines, such as TNF- α, IL-1β□and IL-6, can desensitize bone marrow erythroid progenitors to erythropoiesis, inhibit red blood cell maturation and thereby promote anisocytosis [15, 16].

Though the causative mechanisms of ISR have not yet been fully elucidated, the inflammation is believed to play a pivotal role in the development of ISR. The vascular injuries caused by PCI can up-regulate inflammatory responses and measurements of several factors in blood samples. According to Hojo Y et al., IL-6 concentrations in coronary sinus blood increased immediately after PCI, and there was a positive correlation between increased IL-6 concentrations immediately after PCI and restenoses 6 months after PCI [17].

In our study, the RDW as a marker of inflammatory status was found to increase significantly during follow-up in both ISR and non-ISR groups (Table 2), which was consistent with the up-regulated inflammatory status caused by PCI.

The inflammatory responses to the endothelial denudation and subintimal hemorrhage caused by PCI result in several proliferative processes, including vascular smooth muscle cells proliferation and migration, extracellular matrix formation, and neo-intimal hyperplasia, which are the major pathophysiological mechanisms of ISR [18, 19]. Some physiological or pathophysiological reactions (such as activation of platelets and related growth factors, pro-inflammatory cytokines, leukocytes, and the coagulation–fibrinolysis system, as well as events at the platelet surface) set this proliferative process into motion [20]. Although the inflammatory reactions caused by PCI can be physiological, in some patients the inflammatory reactions may over-response resulting in an overshot proliferative process which can cause ISR of the treated vessels. In our study, the patients with ISR had higher baseline RDWs and follow-up RDWs than the patients without ISR (Table 1), which suggests the patients with ISR may be in a higher level of inflammatory status and have a greater inflammatory response to PCI than those without ISR.

Kuwano T. et al. [21], demonstrated that the baseline serum bilirubin, as an antioxidant agent, could act as an independent predictor of coronary ISR. In our study, both indirect and direct bilirubin were significantly lower in patients who developed ISR (Table 1). We found that only the baseline serum indirect bilirubin was strongly associated with coronary ISR at follow-up and might be an independent predictor of coronary ISR by multivariate logistic regression analysis (Table 5).

## Limitations

In the present study, there were some limitations. First, the sample size was relatively small and study design was retrospective. Large prospective trials are needed to confirm whether RDW can act as a clinical independent predictor of ISR. Second, the late catch-up phenomenon of restenosis especially for DESs beyond 1 year after initial PCI has been recognized [22]. The follow-up periods of 11.1 ± 5.8 months were relatively short and studies with long-term follow-up are required.

## Conclusions

The baseline RDW was closely associated with in**-**stent restenosis at follow-up. The patients with higher baseline RDWs together with lower indirect bilirubin and diabetes had a higher risk of suffering ISR. These findings have important clinical implications. Special attentions should be paid to the patients with higher RDWs before PCI who may have more chances to develop ISR in the future.

## Data Availability

The data and material that support the findings of this study are available in the hospital information system (HIS) of our hospital.

## Abbreviations

DES: Drug-eluting stents
IL: Interleukin
ISR: In-stent restenosis
PCI: Percutaneous coronary intervention
RDW: Red blood cell distribution width
ROC: Receiver operating characteristics
TNF: Tumor necrosis factor
